# Structural basis of Janus kinase *trans*-activation

**DOI:** 10.1016/j.celrep.2023.112201

**Published:** 2023-03-02

**Authors:** Nathanael A. Caveney, Robert A. Saxton, Deepa Waghray, Caleb R. Glassman, Naotaka Tsutsumi, Stevan R. Hubbard, K. Christopher Garcia

**Affiliations:** 1 Department of Molecular and Cellular Physiology, Stanford University School of Medicine, Stanford, CA 94305, USA; 2 Howard Hughes Medical Institute, Stanford University School of Medicine, Stanford, CA 94305, USA; 3 Program in Immunology, Stanford University School of Medicine, Stanford, CA 94305, USA; 4 Department of Biochemistry and Molecular Pharmacology, New York University Grossman School of Medicine, New York, NY 10016, USA; 5 Present address: Department of Molecular and Cell Biology, University of California at Berkeley, Berkeley, CA 94720, USA; 6 These authors contributed equally; 7 Lead contact

## Abstract

Janus kinases (JAKs) mediate signal transduction downstream of cytokine receptors. Cytokine-dependent dimerization is conveyed across the cell membrane to drive JAK dimerization, *trans*-phosphorylation, and activation. Activated JAKs in turn phosphorylate receptor intracellular domains (ICDs), resulting in the recruitment, phosphorylation, and activation of signal transducer and activator of transcription (STAT)-family transcription factors. The structural arrangement of a JAK1 dimer complex with IFNλR1 ICD was recently elucidated while bound by stabilizing nanobodies. While this revealed insights into the dimerization-dependent activation of JAKs and the role of oncogenic mutations in this process, the tyrosine kinase (TK) domains were separated by a distance not compatible with the *trans*-phosphorylation events between the TK domains. Here, we report the cryoelectron microscopy structure of a mouse JAK1 complex in a putative *trans*-activation state and expand these insights to other physiologically relevant JAK complexes, providing mechanistic insight into the crucial *trans*-activation step of JAK signaling and allosteric mechanisms of JAK inhibition.

## In brief

Janus kinases (JAKs) mediate cytokine signaling through dimerization and activation of their kinase domains. Caveney et al. use cryo-EM and AI-guided modeling to reveal the *trans*-activation mechanism of JAKs and allosteric mechanisms of JAK inhibition. This provides the basis for further inhibitor development and insight into the fundamental mechanisms of JAK signaling.

## Graphical abstract

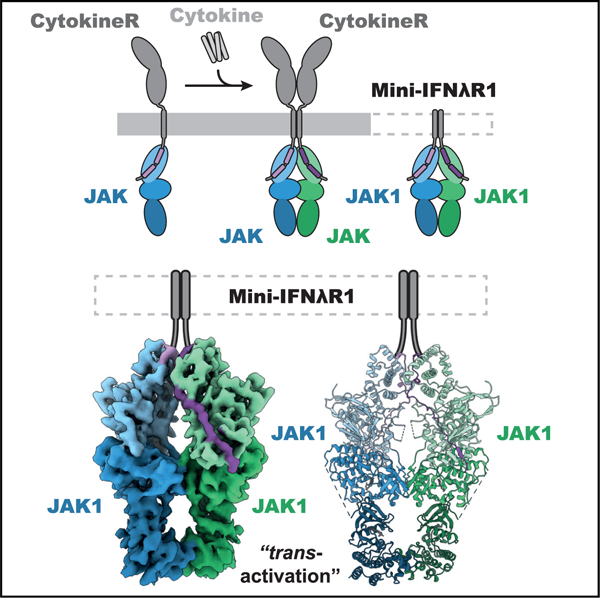

## INTRODUCTION

A large host of cytokines and hormones, including interleukins, interferons, growth hormones, leptin, and erythropoietins, transmit their signals via the Janus kinase (JAK)-signal transducer and activator of transcription (STAT) pathway.^1^ JAKs are protein tyrosine kinases (PTKs) that mediate signal transduction downstream of cytokine receptors.^2–5^ The mammalian JAK family consists of JAK1, JAK2, JAK3, and TYK2, all of which associate with the membrane-proximal regions of cytokine receptors through conserved intracellular domain (ICD) motifs termed “Box1” and “Box2.”^6^ Ligand-dependent dimerization of cytokine receptors is conveyed across the cell membrane to drive JAK dimerization, overcoming an autoinhibitory interaction within the JAK monomer.^7,8^ Dimerization of JAKs enables the *trans*-phosphorylation of the activation loop/segment in the tyrosine kinase domain of JAKs, which stimulates the catalytic activity of the kinase domain. Activated JAKs phosphorylate the ICDs of receptors with which they are associated, leading to recruitment of members of the STAT family, which are in turn phosphorylated by the JAKs prior to transiting to the nucleus to initiate transcription of cytokine-responsive genes.^9^

JAK family members consist of a four-point-one, ezrin, radixin, moesin (FERM) domain, a Src homology 2 (SH2)-like domain, and both a pseudokinase (PK) domain and a tyrosine kinase (TK) domain.^10^ We have recently revealed the structural arrangement of these domains from electron microscopic analysis of a nanobody-stabilized state of an engineered IFNλR1-mJAK1 system.^8^ Here, we report the cryoelectron microscopy (cryo-EM) structure of a homodimeric mouse JAK1 complex in a putative *trans*-activation state and apply artificial intelligence (AI)-guided modeling as implemented in AlphaFold^11,12^ to expand these insights to other physiologically relevant homo- and heterodimeric JAK complexes. Together, these results reveal the structural mechanism of the crucial *trans*-activation step of JAK signaling and provide insights into the development of JAK inhibitors for the treatment of myeloproliferative neoplasms, chronic inflammation, and autoimmunity.

## RESULTS

### Cryo-EM structure of the active IFNλR1-JAK1complex

**Figure 1.**
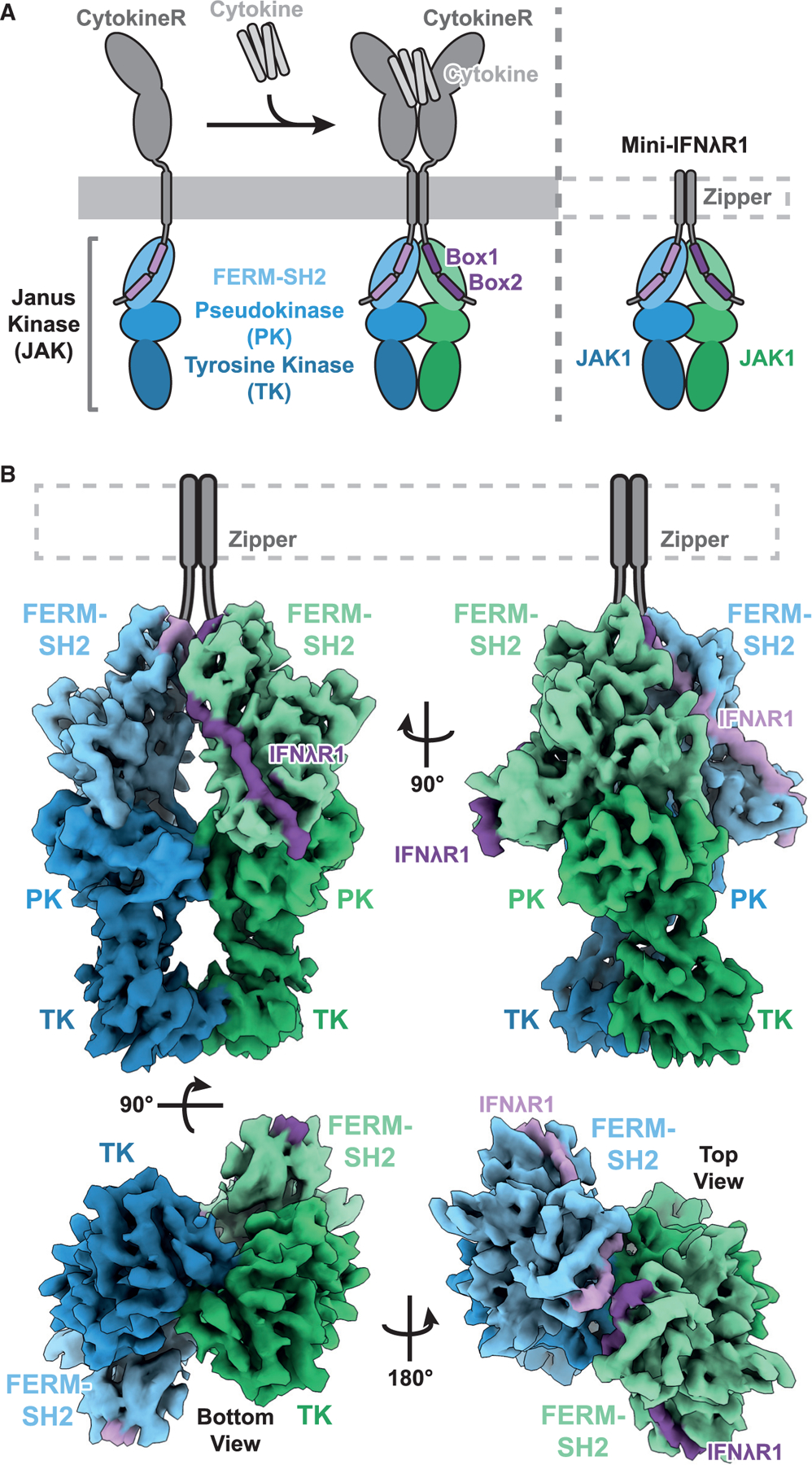
Composition and cryo-EM structure of the trans-activation state JAK complex (A) Cartoon representation of the components of a signaling cytokine receptor complex and the mini-IFNλR1-mJAK1 complex. The two mJAK1 are colored in blue and green, with different shades representing the FERM-SH2, pseudokinase (PK), and tyrosine kinase (TK) domains. Cytokine receptor and mini-IFNλR1 colored in gray and purple. (B) Refined and sharpened cryo-EM density maps of active state mini-IFNλR1-mJAK1 complex, colored as in (A).

**Figure 2.**
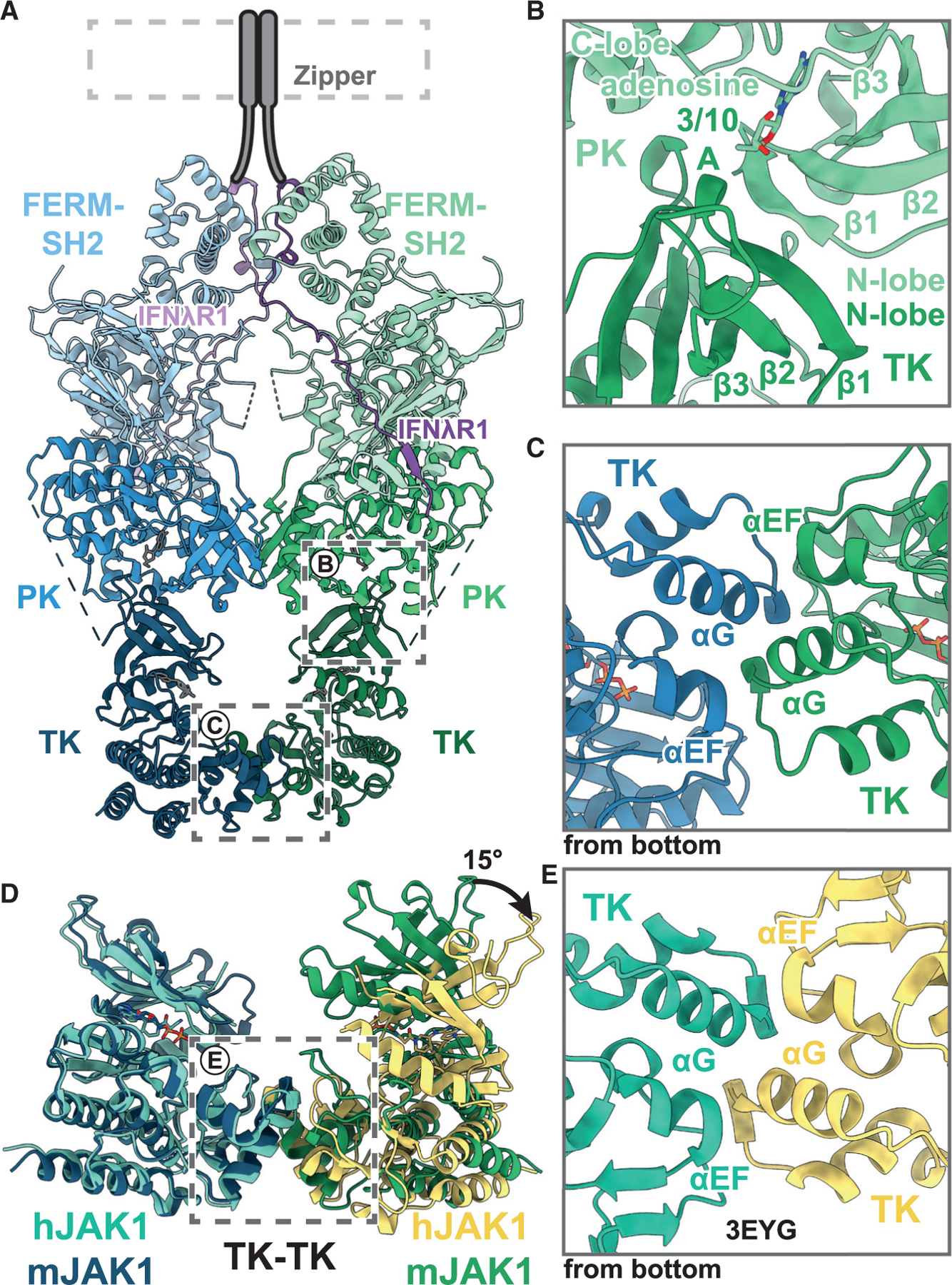
Structure of the active mini-IFNλR1-mJAK1 complex (A) Ribbon representation of a model of mini-IFNλR1-mJAK1 complex in an active conformation. mJAK1 colored in blue and green, with different shades representing the FERM-SH2, PK, and TK domains. Regions highlighted in insets (B) and (C) are boxed in gray. (B) Ribbon representation of the PK-TK interaction interface. (C) Ribbon representation of the TK-TK interaction interface, viewed from the bottom of the complex. (D) A structural overlay comparing mini-IFNλR1-mJAK1 active complex to human JAK1 (hJAK1) TK domain crystal structure (PDB: 3EYG). Mini-IFNλR1-mJAK1 colored as in (A)–(C), and hJAK1 colored in yellow and cyan. (E) Ribbon representation of the TK-TK interaction interface of hJAK1 crystal structure, from viewpoint as in (C) and colored as in (D).

We used the same IFNλR1-mJAK1 system^8^ to reconstitute the intracellular components of a dimerized cytokine receptor-JAK complex, except we did not add the stabilizing nanobodies to the sample prior to imaging in order to attempt to capture a conformation compatible with mJAK1 TK domain *trans*-phosphorylation (Figure 1A). In the absence of the C-terminal TK-stabilizing dimeric nanobodies, we observed particle flexibility that prohibited the generation of *ab initio* models for downstream processing to resolutions compatible with atomistic modeling. Using our reconstruction of the nanobody-trapped state (EMDB-25715), we were able to perform heterogeneous refinement and 3D variability analysis (3DVA) to generate reconstructions of the nanobody-free complex, in which the TK domains are positioned directly below the PK domains. Further refinement generated a 5.5 Å resolution map of a mini-IFNλR1-mJAK1 complex with C2 symmetry (Figures 1B and S1; Table S1). A combination of the nanobody-stabilized complex (PDB: 7T6F) and AlphaFold modeling^11,12^ was used to build an initial model, which was then subject to manual building and refinement, resulting in an atomistic model of the putative *trans*-activation state of the dimeric mini-IFNλR1-mJAK1 complex (Figure 2A).

As in the nanobody-stabilized complex, in the nanobody-free complex, we observe a similar arrangement of both the membrane-proximal FERM-SH2-INFλR1 and the dimerized PK domain below, additionally stabilized by the V685F mutation. These two domains form a compact unit with clear density and an absence of significant interdomain flexibility (Figure 1). In the nanobody-free state, we observe that the TK domains rotated down and inward from the outward-facing state observed previously^8^ (Figure S2), forming a small interaction interface that orients the kinase active sites toward one another in a pose compatible with *trans*-phosphorylation of the activation loops, which we deem the *trans*-activation state.

### JAK1 kinase interfaces and structural conservation

The PK and TK domains of JAKs each possess the canonical eukaryotic kinase bilobed structure comprising a smaller N-terminal lobe (N lobe) that consists of a twisted five-stranded β-sheet (β1–5) flanked by a lone α-helix (αC) and a larger C-terminal lobe (C lobe) that is dominated by α-helical structure (αD–I) and includes the catalytic and activation loops. ATP binds in the cleft between the two lobes. In the TK-PK pose observed in the nanobody-free mini-IFNλR1-mJAK1 complex, the β-sheet from the N lobe of the TK domain abuts the ATP-binding pocket of the PK domain, interacting with residues in both the N and C lobes of the PK domain (Figure 2B). In the short 3–10 helix that precedes β1 in the TK domain, Arg872 (mJAK1) is within predicted salt-bridging distance to Asp675 in αD (C lobe) of the PK domain. These two residues are strictly conserved in the JAK family. The total surface area buried in the mJAK1 PK-TK interface is relatively small (584 Å^2^, per AlphaFold-guided modeling into the observed density), consistent with inherent flexibility in this region as observed in the cryo-EM data.

At the TK-TK domain interface, a primarily α-helical interaction is formed (Figure 2C). This interaction is largely composed of the helical packing contributions from αG and αEF. The respective αG from each monomer packs against the other, while the C-terminal end of αEF abuts the packing interface. As in the PK-TK interface, the TK-TK interface provides little buried surface area (783 Å^2^, ~5% of TK surface area, per AlphaFold-guided modeling into the observed density), in line with the proposed transient nature of this interaction, which is likely necessary for JAK-mediated receptor and STAT phosphorylation.

Interestingly, the TK-TK interface observed in this work bears striking similarity to a crystallographic interface that is observed in multiple structures of the kinase domain of human JAK1^13^ (Figures 2C and 2D). In both the crystallographic structures and the present cryo-EM structure, α-helices G and EF form the bulk of the interface (Figure 2D). In the case of the crystallographic TK arrangement, there is an approximate 15° outward rotation of the N and C lobes, which may be due to the TK domain not being constrained by the PK domain in the context of the crystallographic construct (Figure 2C).

### AlphaFold structural predictions for JAK homo- and heterodimers

AlphaFold predictions^11^ of the monomeric structures of the four JAK proteins exhibit two distinct configurations: one in which the PK-TK interaction is similar to that observed in the crystal structure of TYK2 PK-TK,^7^ representing the autoinhibited state of a JAK, and one in which the PK-TK interaction highly resembles the *trans*-activation state of the present cryo-EM structure of homodimeric mJAK1 (Figure S3).

We sought to assess the generality of our JAK1 structure to other JAK dimers found in nature. Based on the AlphaFold predictions of JAK monomers and the dimeric mJAK1 cryo-EM structure, we hypothesize that *trans*-activation of all JAKs, in the various homo- and heterodimeric configurations in which they occur, utilizes the same PK-TK dimeric unit observed in the present mJAK1 cryo-EM structure. We further hypothesize that this PK-TK dimeric unit is the functional structural entity whether the PK domain harbors an activating mutation (such as V617F in JAK2 or V657F in mJAK1) and dimerization is cytokine independent or whether cytokine-induced dimerization juxtaposes two wild-type JAKs.

To provide support for this hypothesis, we used AlphaFold^11,12^ to predict PK-TK dimeric structures for the various physiologic, wild-type JAK dimers: JAK2-JAK2, JAK1-JAK2, JAK1-JAK3, JAK1-TYK2, and JAK2-TYK2 (Figure S3). For each JAK PK-TK dimer, between five and ten models were generated. For most of the PK-TK dimers, the highest-scoring model exhibited the same PK-TK (*cis*), PK-PK (*trans*), and TK-TK (*trans*) interfaces as those observed in the mJAK1 cryo-EM structure.

**Figure 3.**
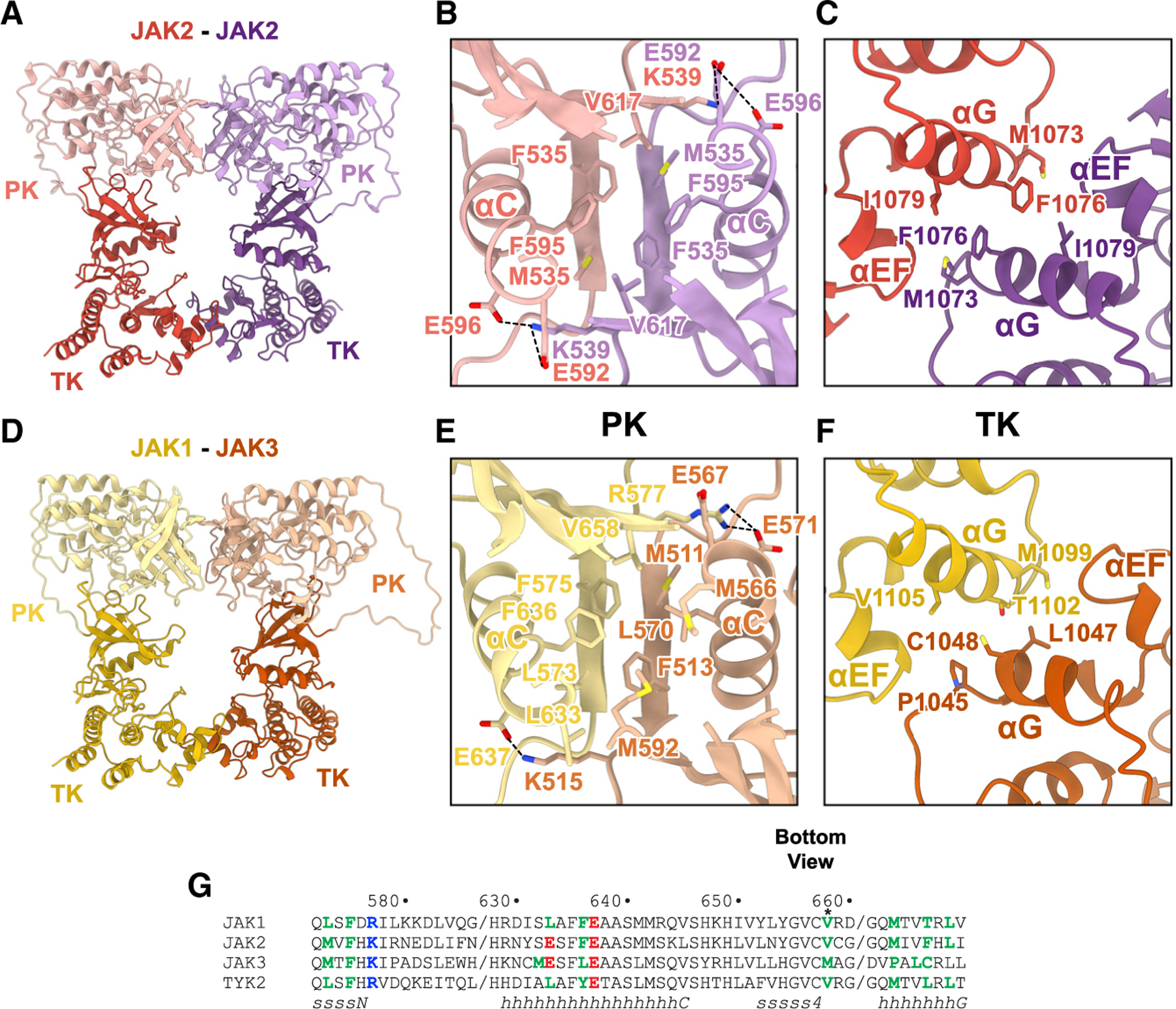
AlphaFold predictions for JAK homo- and heterodimers (A) Ribbon representation of a prediction of the PK and TK domains of a human JAK2 homodimer. JAK2 in red and purple, with lighter and darker coloring for PK and TK, respectively. (B) PK interface of the JAK2 homodimer, colored as in (A). Predicted salt bridges depicted with dashed lines. (C) TK interface of the JAK2 homodimer, colored as in (A). (D) Ribbon representation of a prediction of the PK and TK domains of a human JAK1-JAK3 heterodimer. JAK1 in yellow and JAK3 in orange, with lighter and darker coloring for PK and TK, respectively. (E) PK interface of the JAK1-JAK3 heterodimer, colored as in (D). (F) TK interface of the JAK1-JAK3 heterodimer, colored as in (D). Predicted salt bridges depicted with dashed lines. (G) Sequence alignment of key dimerization regions of human JAK1, JAK2, JAK3, and TYK2. Key residues in bold, with basic residues in blue, acidic residues in red, and hydrophobic residues in green. mJAK1 V657F activating mutation marked by an asterisk (*), showing conservation of hydrophobicity at this position among all JAKs.

For homodimeric JAK2 PK-TK (Figures 3A–3C), the symmetric PK-PK interface predicted by AlphaFold comprises four hydrophobic residues from each protomer: Met535 and Phe537 from the β-strand N-terminal to the start of the PK domain, Phe595 in αC, and Val617 (site of V617F activating mutation) at the end of β4. This hydrophobic interface is fortified at the periphery by predicted salt bridging between Lys539, at the beginning of the PK domain, and Glu592 and Glu596 in αC. Glu596 is present in all four JAKs, whereas Glu592 is found in JAK2 and JAK3, with leucine present in JAK1 (Leu633) and TYK2 (Figure 3G). E596R and E592K have been shown to be loss-of-function mutations, whereas K539L and E592W are gain-of-function mutations.^2,14^

In the asymmetric PK-PK interface for the JAK1-JAK3 heterodimer predicted by AlphaFold (Figures 3D–3F), residues in the same positions as in the JAK2 homodimer contribute (Figurer 3E), with the following differences. Met592 in JAK3 replaces Val617 in JAK2 (M592F is not activating), Leu633 in JAK1 (αC) provides an additional hydrophobic contact, and Glu567 and Glu571 (αC) in JAK3 are salt bridged to Arg577 in JAK1. JAK3 contains a unique hydrophobic residue in αC, Met566 (Figure 3G), which also contributes to the dimerization interface.

The TK-TK interface predicted by AlphaFold for the JAK2 homodimer and for the assorted JAK heterodimers highly resembles that observed in the mJAK1 cryo-EM structure, with hydrophobic residues in αG underpinning the interface. For the JAK2 homodimer (Figure 3C), residues Met1073, Phe1076, and I1079 in αG form a small hydrophobic cluster with their symmetric counterparts. The I1079 position in PTKs is typically hydrophobic—involved in C-lobe helix interactions—but Met1073 and Phe1076 are largely solvent exposed in a JAK2 monomer, and Phe1076 is typically hydrophilic in PTKs. Met1073 is at the beginning of an N-terminally extended (relative to other PTKs) αG that follows a JAK-specific helical insertion. The TK-TK interface predicted for the JAK1-JAK3 heterodimer consists of Met1099, Thr1102, and Leu1105 in αG of JAK1 and Pro1045, Leu1047, and Cys1048 in αG of JAK3 (Figure 3F). Leu1047 is unique to JAK3; the smaller valine is at this position in the other JAKs.

Evidently, all pairings of JAKs can be accommodated in the PK-TK dimeric unit observed in the mJAK1 cryo-EM structure and predicted by AlphaFold. Thus, the actual physiologic pairings are presumably dictated solely by differential JAK-cytokine receptor associations and the type and number of receptor chains that a cytokine assembles. This is consistent with previous work showing that the non-physiological JAK2/JAK3 dimer is signaling competent.^15^

### Flexibility of the TK domains of JAK1

**Figure 4.**
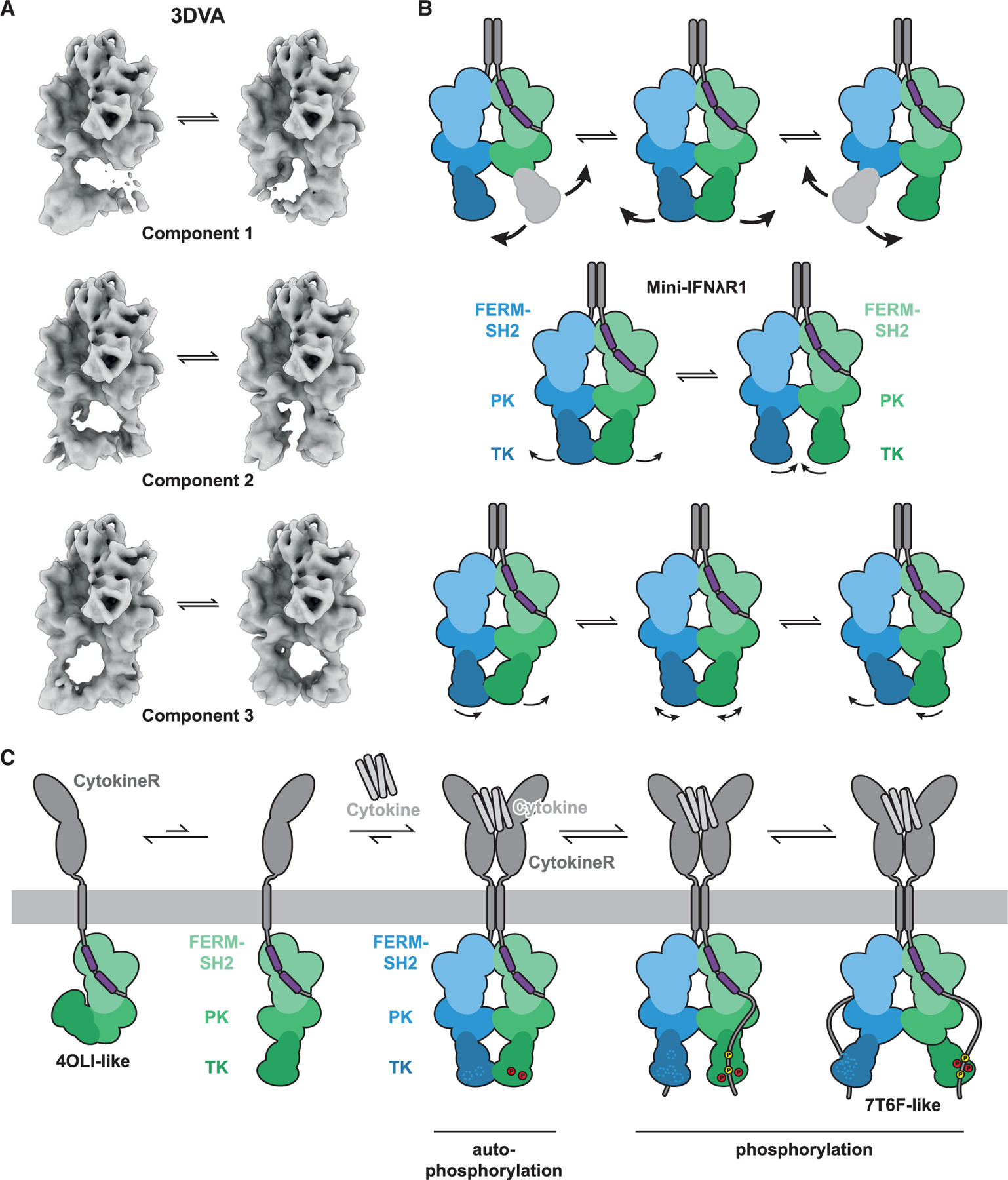
Structural dynamics of the mini-IFNλR1-mJAK1 complex (A) 3D variability analysis of the kinase domains of the mini-IFNλR1-mJAK1 complex. Variability components with significant movement and reorientation are shown with their representative initial and final reconstructions. (B) Schematic representations of the movements depicted in (A). Mini-IFNλR1-mJAK1 colored in gray and purple, and mJAK1 colored in blue and green. (C) A schematic representation of the role of the observed flexibility in activation of a cytokine receptor complex. Colored as in (B), with *trans*-phosphorylation of the TK activation loop represented in red and receptor phosphorylation represented in yellow.

Given the difficulties in obtaining a stable reconstruction of the active IFNλR1-mJAK1 complex, we explored the potential flexibility of the complex using 3DVA. Using a wide mask for global analysis of flexibility, we identified no significant movement across the dimer of FERM-SH2 and PK domains. Therefore, subsequent analysis was performed with a generous mask focused on the TK domains in order to specifically explore flexibility in this region (Figure 4).

In our 3DVA, we report three components of variability in the TK domain density (Figures 4A and 4B). One of the most significant components involved the complete loss of density in the TK domain of one monomer or the other. We interpret this to represent a highly motile state for the TK domain free of distinct substates along the path of motion that would resolve into visible density in this analysis. This apparent flexibility is consistent with the distance that the TK domains must travel between the previously reported nanobody-trapped state and the activation-competent state resolved in this work. In addition to this component with a large amount of variability, there are two components resolved that consist of smaller movements in the TK domains (Figures 4A and 4B). These components exhibit lateral movements of the TK domain that are distinguished by occurring in opposition or conjunction across the dimer interface. In the first component, the domains move in opposition, flexing outward and releasing contact with one another at the TK-TK interface. In the second, these movements occur in conjunction, and a shuffling of the TK domains laterally in either direction is observed, with no release at the TK-TK interface.

## DISCUSSION

### JAK *trans*-activation

The present cryo-EM structure of IFNλR1-mJAK1 depicts two JAK1 molecules symmetrically dimerized via the N lobe of the PK domain, with the TK domains interacting through their C lobes and their active sites facing one another (Figure 2A). This *trans*-activation pose is the state through which all dimerized JAKs, as either homodimers (JAK1, JAK2) or heterodimers (all JAKs), will undergo *trans*-phosphorylation of the activation loop. All JAKs possess two tyrosine residues in the activation loop (Tyr1033/1034 in mJAK1), the phosphorylation of which stabilizes a conformation that is optimized for substrate binding and catalysis.^16^ Activation-loop phosphorylation in the TK domain is the critical first step in JAK activation and subsequent downstream phosphorylation events.

Due to inherent flexibility of the JAK TK domain in our dataset, this region is not resolved at a resolution sufficient to accurately trace the conformation of the phosphorylated activation loops. The density best supports modeling of this loop in a conformation similar to that observed in both phosphorylated and unphosphorylated JAK1 TK crystal structures (e.g., 4OLI, 3EYG^7,13^). However, rudimentary modeling (Figure S4) suggests that, in this TK-TK configuration, the tyrosine residues in the activation loop of one TK domain are capable of reaching the active site of the opposing TK domain to be phosphorylated. Interestingly, in contrast to the majority of protein kinases, crystal structures have revealed that the unphosphorylated activation loop in JAK TK domains adopts a conformation similar to that of the phosphorylated activation loop. Whether phosphorylation of the activation loop destabilizes the TK-TK *trans*-activation pose to favor phosphorylation of other sites (e.g., receptor ICD) by the TK domain, however, remains to be determined.

In the context of our previously proposed model for the activation of JAKs,^8^ the flexibility of the TK domain relative to the rest of the molecule would be necessary for proper regulation and function. In the basal, unstimulated, state, the activity of the TK domain is inhibited through an interaction (in *cis*) with the PK domain.^7,17^ Upon receptor dimerization, homotypic interactions between adjacent PK domains relieve autoinhibition to enable *trans*-phosphorylation of the activation loop and subsequent phosphorylation of both the receptor and receptor-bound STATs. The range of motion required in this scenario is vast and is largely captured between the structures of occluded monomeric TYK2 PK-TK and our nanobody-stabilized states, with the conformation presented in this work likely being an intermediate state, both within the context of JAK flexibility and its mechanistic function (Figure 4C). The flexibility observed for the putative active JAK complex, between both the nanobody-stabilized state and the *trans*-activation state observed here, is therefore likely required for engaging distal tyrosine residues on the receptor ICD and on receptor-bound STATs, which are often located many residues apart from one another.

### Allosteric JAK inhibition via the PK domain

**Figure 5.**
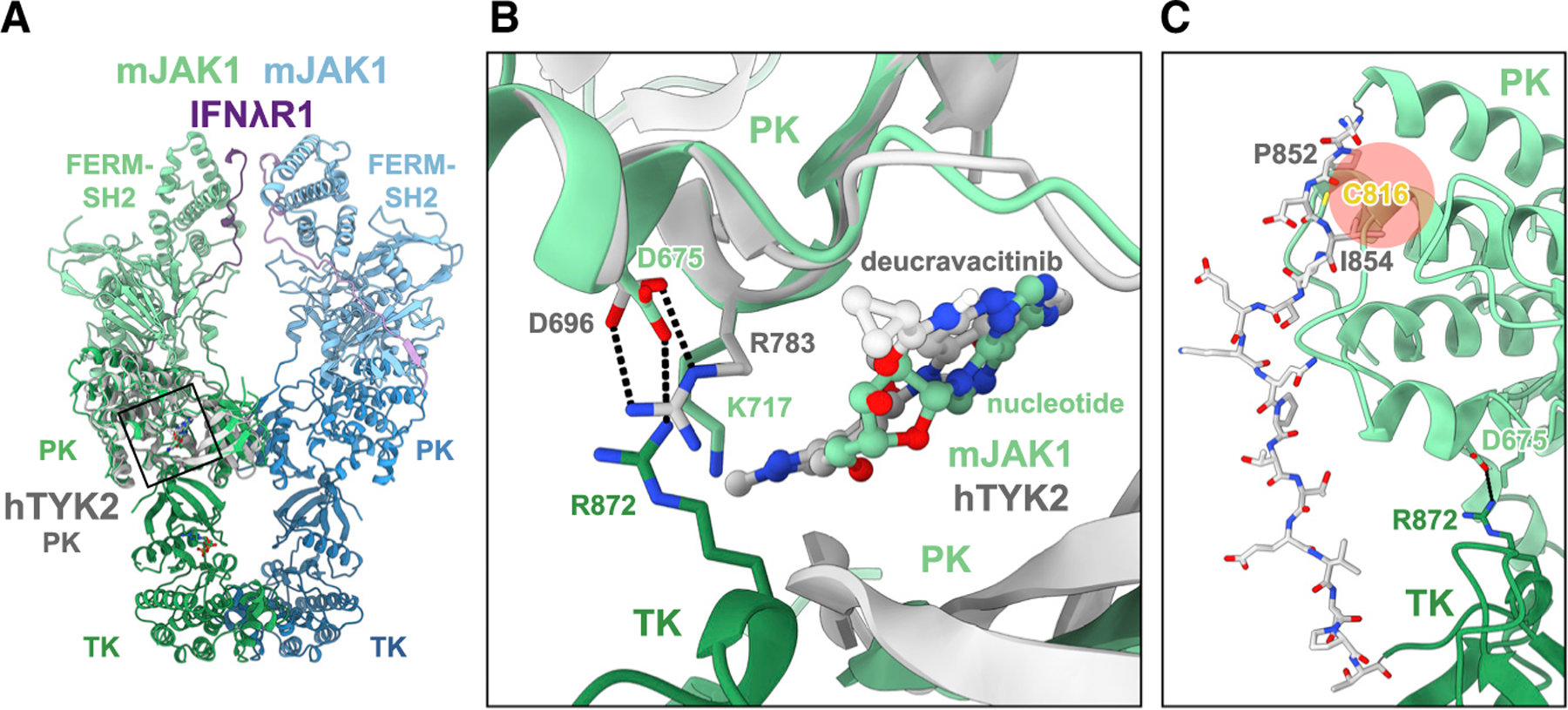
Allosteric JAK inhibition via the PK domain (A) Structural overlay of the mouse JAK1 cryo-EM structure and human TYK2 PK with bound deucravacitinib (PDB: 6NZP). mJAK1 in green and blue, IFNλR1 in purple, and hTYK2 in gray. (B) Structural overlay of the mouse JAK1 cryo-EM structure and human TYK2 PK with bound deucravacitinib. Colored as in (A). Key residues displayed, with mJAK1 *trans*-PK-TK hydrogen bonding, as predicted by AlphaFold guided modeling, and hTYK2 *cis*-PK hydrogen bonding from the crystal structure shown with dashed lines. (C) mJAK1 cryo-EM structure in green with AlphaFold modeled PK-TK linker region in gray. C816, which is modified by the inhibitor VVD-118313, is depicted, and the region the compound would bind is colored in red.

The interaction between the PK and TK domains observed in the current mJAK1 cryo-EM structure, and in the AlphaFold structural models for JAKs, suggests a possible mechanism for the inhibitory action of small molecules that bind in the ATP-binding pocket of the TYK2 PK domain, so-called allosteric inhibitors.^18–20^ These molecules bind specifically to the PK domain of TYK2, not to the TK domain, yet they inhibit TYK2 signaling pathways. In Figure 5B, the crystal structure of BMS-986165 (deucravacitinib) bound to TYK2 PK (PDB: 6NZP)^19^ is superimposed on the mJAK1 cryo-EM structure. In the TYK2 PK domain crystal structure, the JAK-invariant aspartic acid in αD (Asp696) is salt bridged to Arg738 (Lys717 in mJAK1) in the catalytic loop. Formation of this “*cis*” (within PK domain) Arg-Asp salt bridge, which is induced by compound binding, would compete with the “*trans*” (between PK and TK domains) Arg-Asp salt bridge, present in the AlphaFold models of all four JAKs, which we posit to be important for stabilizing the PK-TK *trans*-activation pose (Figure 5B). Thus, we hypothesize that these compounds inhibit TYK2 signaling by preventing efficient TKD *trans*-phosphorylation through disruption of the enabling PK-TK interface.

A second type of JAK PK-binding allosteric inhibitor was recently reported.^20^ This JAK1 inhibitor binds covalently to Cys816 (Cys817 in human JAK1) in the C lobe of the PKD, distal to the ATP-binding pocket, filling a pocket formed by helices αE, αF, αH, and αI. Although the linker between the PK and TK domains is mostly disordered in the mJAK1 cryo-EM structure, in the AlphaFold structural model for full-length JAK1, the PK-TK linker runs along the bottom of the C lobe of the PK domain to the TK domain (Figure 5C). Pro852 in the beginning of the linker is inserted, as is Ile854, in the pocket where the covalent inhibitor will bind. Hydrophobic residues are conserved at these positions in JAK1 and TYK2, which, interestingly, have a PK-TK linker that is fifteen residues shorter than in JAK2 and JAK3. We hypothesize that binding of the covalent inhibitor in this C lobe pocket in the JAK1 PK domain disrupts the conformation of the PK-TK linker, resulting in destabilization of the PK-TK *trans*-activation pose and loss of JAK1-mediated phosphorylation.

Interestingly, a human JAK3 gain-of-function mutation in the PK domain, V722I,^21^ is in the interface between the PK domain and the PK-TK linker, according to the AlphaFold model for JAK3. The larger hydrophobic residue (isoleucine) could potentially hyperstabilize the PK-TK linker and the PK-TK interaction, resulting in a more stable TK-TK *trans*-activation platform. Moreover, Tyr813 in the JAK2 PK-TK linker is a JAK2 auto-phosphorylation site and has been shown to recruit the adapter protein SH21B, which positively regulates JAK2 activity.^22,23^ Whether inhibitor bound, enhancer bound, or mutated, the PK-TK linker region plays a clear role in the allosteric regulation of JAK activity.

The structural insights into the JAK activation process gained through the cryo-EM structures of dimerized mJAK1, both past^8^ and present, and complemented by structural predictions from AlphaFold, should provide new drug discovery opportunities to treat chronic inflammation, autoimmune diseases, myeloproliferative neoplasms, and cancers arising from aberrant activation of JAK-STAT signaling pathways.

### Limitations of study

The current work describes a 5.5 Å resolution structure of the putative *trans*-activation state of the engineered mini-IFNλR1-JAK1 complex. While AI-guided modeling and the previous nanobody-stabilized mJAK complex structure (PDB: 7T6F^8^) allow for high confidence in our model, we acknowledge the limitations imposed at this resolution. Further experimentation may be required to capture this state at a higher resolution, and modifications to the protocols described here may be required to stabilize and capture the intermediate A-loop crossing event. Additionally, the AlphaFold^11^ models of the various additional physiologically relevant JAK dimers could be experimentally validated through an adaptation of the protocols described here.

## STAR★METHODS

### RESOURCE AVAILABILITY

#### Lead contact

Further information and requests for resources and reagents should be directed to and will be fulfilled by the lead contact, KCG (kcgarcia@stanford.edu).

#### Materials availability

The plasmids used in this work are available from the Lead Contact by request.

#### Data and code availability

- CryoEM maps and atomic coordinates for *trans-*activation state mJAK1 complex have been deposited in the EMDB (EMD-28649) and PDB (8EWY) respectively. Models for JAK2-JAK2, JAK1-JAK3, JAK1-JAK2, JAK1-TYK2, and JAK2-TYK2 have been deposited in ModelArchive (ma-evjj8, ma-6l9wz, ma-11w6k, ma-jv4is, ma-usl3x respectively).
- This paper does not report original code.
- Any additional information required to reanalyze the data reported in this work paper is available from the lead contact upon request.

### EXPERIMENTAL MODEL AND SUBJECT DETAILS

For generation of baculovirus, *Spodoptera frugiperda* (*Sf*9) ovarian cells (ATCC) were used. They were cultured in Sf-900 III medium (Gibco) with 10% (v/v) FBS (Sigma) and GlutaMAX (Gibco) at 27°C with ambient CO_2_ and gentle agitation. For protein expression, *Trichoplusia ni* (*T. ni*) ovarian cells (Expression Systems) were used. They were maintained in ESF 921 Insect Cell Culture Medium (Expression Systems) at 27°C with ambient CO_2_ and gentle agitation.

### METHOD DETAILS

#### Cloning and protein expression

Constructs and baculoviruses were used as described previously.^8^ Mini-IFNλR1, consisting of N-terminal glutathione S-transferase followed, 9 amino acid linker (SDGSTSGSG), 3C protease site, GCN4 leucine zipper, and *Mus musculus* interferon lambda receptor 1 (IFNllR1) Box1/Box2 (mIFNλR1 249–298) was cloned into a modified pAc vector for baculoviral expression. Full-length *Mus musculus* Janus Kinase 1 (mJAK1 1–1153) with C-terminal BC2 (DRKAAVSHWQ) and 8xHis tags was cloned into pAc with activating V657F mutation. *Spodoptera frugiperda* (*Sf*9) ovarian cells (ATCC) were cultured in Sf-900 III medium (Gibco) with 10% (v/v) FBS (Sigma) and GlutaMAX (Gibco). Transfection with FuGENE HD (Promega) and BestBac 1.0 Linearized Baculovirus DNA (Expression Systems) was used to produce Baculovirus. Protein was expressed in *Trichoplusia ni* (*T. ni*) ovarian cells (Expression Systems) maintained in ESF 921 Insect Cell Culture Medium (Expression Systems) at 27°C with ambient CO_2_ and gentle agitation. *T. ni* cells were infected with both mini-IFNλR1 and mJAK1 baculovirus for 48 hours. Cells were washed in Phosphate Buffered Saline (PBS) pH 7.4 prior to freezing for storage.

#### Protein purification

Cells were resuspended in 50 mM Tris-HCl pH 8.5, 0.5 M NaCl, 1 mM adenosine (Sigma), 1 mM TCEP, 10% (v/v) glycerol, 15 mM imidazole, protease inhibitor cocktail (Sigma), and benzonase (Sigma). Cells were lysed by Dounce homogenization and cellular debris was pelleted by ultracentrifugation. The soluble fraction was subject to affinity purification using High Affinity Ni-Charged Resin (GenScript) in lysis buffer supplemented with 0.005% (w/v) n-dodecyl β-D-maltoside (DDM). Nickle resin was washed with buffer additionally supplemented with 30 mM imidazole and subsequently eluted with 250 mM imidazole. The elution was then incubated with Glutathione Sepharose 4 Fast Flow Resin (Cytoiva), washed in 20 mM HEPES-Na pH 8.0, 0.5 M NaCl, 1 mM adenosine, 1mM TCEP, 10% (v/v) glycerol, and 0.005% (w/v) DDM, and eluted in buffer supplemented 20 mM reduced glutathione and adjusted to pH 7.4. The elution was then concentrated and buffer exchanged into buffer free of reduced glutathione prior to cleavage with 1:10 (w/w) 3C protease for 1 hour at room temperature. Superose 6 (Cytiva) SEC was used to separate mJAK1-IFNλR1 complex from free GST in 20 mM HEPES-Na pH 8.0, 0.5 M NaCl, 1 mM adenosine, 1% (v/v) glycerol, and 1 mM TCEP. Purified sample was crosslinked with 1 mM bis(sulfosuccinimidyl)suberate (BS3) (Thermo) for 30 min at room temperature and quenched with 20 mM Tris-HCl pH 8. The cross-linked complex was buffer-exchanged into 20 mM HEPES-Na pH 8, 0.5 M NaCl, 1 mM adenosine, 1% (v/v) glycerol, and 1 mM TCEP on a Vivaspin 500 centrifugal concentrators (Satorius, 100,000 MWCO) and concentrated to 1 mg/ml for cryo-EM experiments.

#### Cryoelectron microscopy

Aliquots of 3 μL of complex were applied to glow-discharged Quantifoil^®^ (1.2/1.3) grids. The grids were blotted for 3 seconds at 100% humidity with an offset of 3 and plunge frozen into liquid ethane using a Vitrobot Mark IV (Thermo Fisher). Grids were imaged on a 300 kV FEI Titan Krios microscope (Thermo Fisher) located at the Stanford cEMc facility and equipped with a K3 camera and energy filter (Gatan). Movies were collected at a calibrated magnification of ×130,000, corresponding to 0.653 Å per physical pixel. The dose was set to a total of 50.6 electrons per Å^2^. Automated data collection was carried out using SerialEM^24^ with a nominal defocus range set from −0.8 to −3.0 μM. 21,154 movies were collected to compensate for low particle density on the imaged grids.

#### Image processing

All processing was performed in cryoSPARC^25^ unless otherwise noted (Figure S1). The 21,154 movies were motion corrected using patch motion correction. The contrast transfer functions (CTFs) of the flattened micrographs were determined using patch CTF and an initial stack of particles was picked using Topaz picker.^31^ Successive rounds of 3D heterogenous refinement and 3DVA^32^ using the nanobody-stabilised mJAK1 complex (EMD-25715) both with and without trimmed TK domain density, were used to pull out a low-resolution reconstruction with TK density directly below the FERM-SH2 and PK domains, specifically with contact between the two TK domains. This model was then used in 4 rounds of 3D heterogenous refinement, with C2 symmetry enforced, to generate a reconstruction using 174,962 particles. This was subsequently used in non-uniform refinement^33^ and local refinement with a loose mask around the entire complex. This resulted in a reconstruction with a resolution of 5.5 Å. Resolution was determined at a criterion of 0.143 Fourier shell correlation gold-standard refinement procedure. This reconstruction was then used in 3DVA^32^ with a lowpass filter of 12 Å and a loose mask around the TK domains and the lower portions of the PK domains. 3DVA was run using a global mask, though no significant movement was seen outside of the TK domains.

#### Model building and refinement

The nanobody stabilised mJAK complex structure (PDB 7T6F) and AlphaFold models^11,12^ were docked into the map using UCSF Chimera X.^27^ The resultant model was then refined using Phenix real space refine^28^ and manual building in Coot.^29^ The final model produced a favorable MolProbity score of 1.26^30^ with 94.78% Ramachandran favoured and 0.09% outliers (Table S1).

#### AlphaFold modeling of JAK PK-TK homo- and heterodimers

AlphaFold as implemented in Google ColabFold notebook AlphaFold2_advanced (Mirdita et al. 2022) was used to generate atomic models for the JAK2 PK-TK homodimer and for PK-TK heterodimers of various JAKs (JAK1-JAK2, JAK1-JAK3, JAK1-TYK2, and JAK2-TYK2). This ColabFold notebook runs AlphaFold2 without structural templates and uses mmseq2 for performing multiple sequence alignments. The human sequences for JAK1–3 and TYK2 were input, starting in the linker between the SH2 and PK domains, approximately 15 residues upstream of the β-strand just N-terminal to the PK domain (see Figure 3G), and ending at the C-terminus of the protein. For each run, either five models (num_models=5, num_samples=1) (all JAK combinations except JAK1-JAK3) or ten models (num_models=5, num_samples=2) (JAK1-JAK3) for dimeric PK-TK were generated, ranked by pTMscores. The PK-TK dimeric configuration observed in the cryoEM map for nanobody-free mJAK1 was the highest scoring model for all but the JAK1-JAK3 combination, for which it was the third-highest scoring model (of ten). Each of the PK-TK homo- or heterodimers was subject to geometry minimization in PHENIX,^28^ employing the default parameters.

For modeling of the activation loop of JAK1 in the active site of JAK3, the AlphaFold model for the JAK1-JAK3 PK-TK heterodimer was used as the starting point. The crystal structure of a substrate peptide bound to the insulin receptor kinase domain (PDB code 1IR3) was superimposed on the catalytic loop of JAK3. The tyrosine of the substrate peptide from the 1IR3 structure, along with the residues on either side of the tyrosine became residues 1033–1035 (EYY) of the JAK1 activation loop, with the rest of the JAK1 activation loop from the AlphaFold model deleted. This incomplete PK-TK heterodimer was the input to Swiss-Model,^26^ which constructed the remainder of the JAK1 activation loop. The completed model was then subjected to geometry minimization in PHENIX.^28^

## Supplementary Material

1

## Figures and Tables

**Table:** 

REAGENT or RESOURCE	SOURCE	IDENTIFIER
Deposited data		

CryoEM map *trans-*activation state mJAK1 complex	EMDB	EMD-28649
Atomic coordinates for *trans-*activation state mJAK1 complex	PDB	8EWY
JAK1 kinase domain	PDB	3EYG
Pseudokinase/kinase domains of TYK2	PDB	4OLI
TYK2 with deucravacitinib	PDB	6NZP
JAK2-JAK2 PK-TK model	ModelArchive	evjj8
JAK1-JAK3 PK-TK model	ModelArchive	6l9wz
JAK1-JAK2 PK-TK model	ModelArchive	11w6k
JAK1-TYK2 PK-TK model	ModelArchive	jv4is
JAK2-TYK2 PK-TK model	ModelArchive	usl3x

Experimental models: Cell lines		

cell line (*Spodoptera frugiperda*)	ATCC	*Sf9*
cell line (*Trichoplusia ni*)	Expression Systems	94–002F

Recombinant DNA		

pAc_mini-IFNλR1 (plasmid)	Glassman et al.^8^	N/A
pAc_mJAK1_V657F (plasmid)	Glassman et al.^8^	N/A

Software and algorithms		

SerialEM – Data collection software	Mastronarde^24^	SerialEM 4.0
cryoSPARC – Data processing software	Structura Biotechnology Inc. Punjani et al.^25^	cryoSPARC v4.0.1
AlphaFold – Modelling software	Jumper et al.^11^	N/A
ColabFold – Modelling software	Mirdita et al.^12^	N/A
SWISS-MODEL – Modelling software	Waterhouse et al.^26^	N/A
UCSF ChimeraX – Graphics software	Pettersen et al.^27^	N/A
Phenix – Modelling and refinement software	Adams et al.^28^	N/A
Coot – Modelling and refinement software	Emsley et al.^29^	N/A
MolProbity – Model validation software	Chen et al.^30^	N/A

## Highlights

- Cryo-EM structure of the putative Janus kinase *trans*-activation state
- Tyrosine kinase domains rotate downward to autophosphorylate
- Artificial intelligence-guided modeling of other physiological JAK complexes
- Insight into allosteric mechanisms of JAK inhibition
