# Supplementary material for: Structural basis of Janus kinase trans-activation
Source: Cell Rep. Author manuscript; Available in PMC 2023 May 12. (PMC10180219; doi:10.1016/j.celrep.2023.112201)
Supplement: 1 [file NIHMS1887282-supplement-1.pdf]

Cell Reports, Volume *42*

Supplemental information

Structural basis of Janus kinase *trans*-activation

Nathanael A. Caveney, Robert A. Saxton, Deepa Waghray, Caleb R. Glassman, Naotaka Tsutsumi, Stevan R. Hubbard, and K. Christopher Garcia

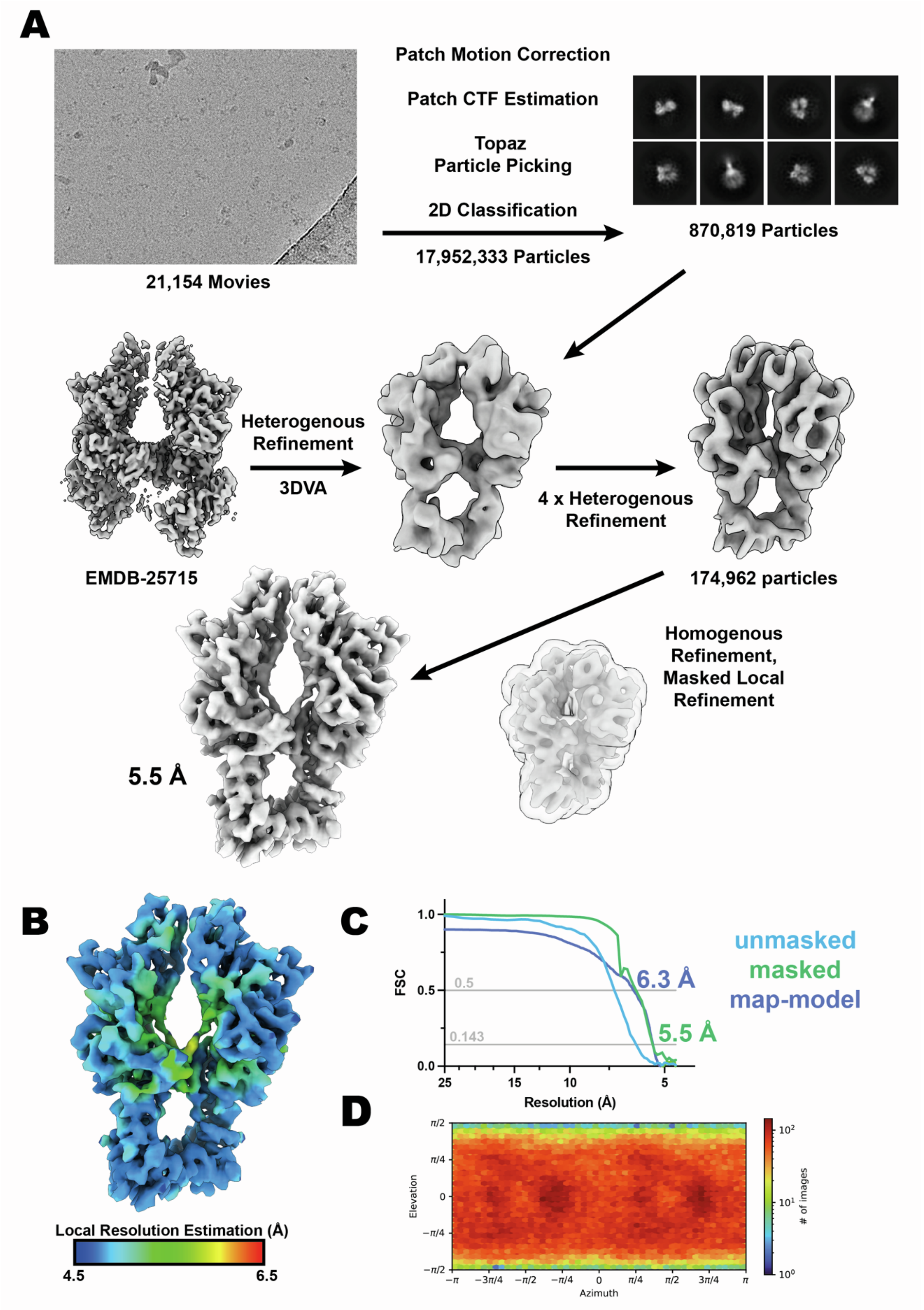

Figure S1. JAK1 complex cryoEM data processing, Related to Figure 1 and STAR Methods. (**A**) Workflow for cryoEM data processing. Representative micrograph, reference free 2D averages, and cryoEM maps at the various stages of processing. (**B**) Local resolution estimation of the finalised cryoEM map [S1]. (**C**) FSC curve of the reconstruction using gold-standard refinement calculated from unmasked and masked half maps. Map-model FSC curve. (**D**) Orientational distribution of the reconstruction.

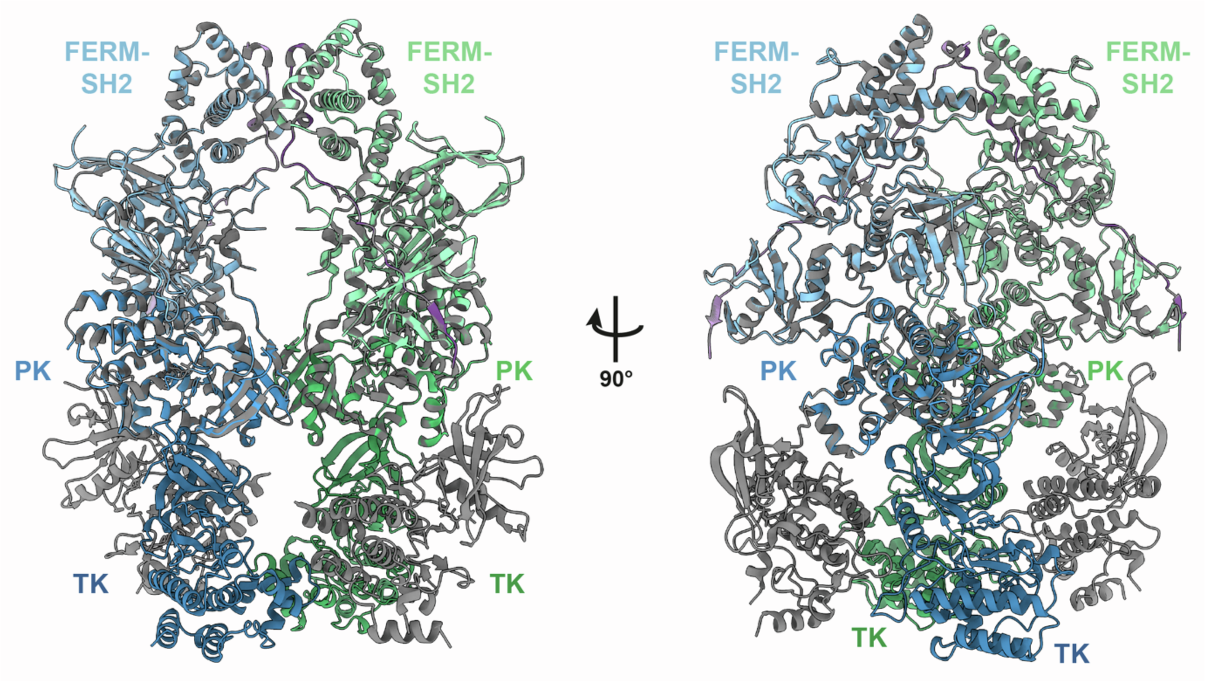

Figure S2. Structural comparison of JAK1 complex with and without nanobody stabilization, Related to Figure 2. Overlay of a ribbon representation of the nanobody-free JAK1 complex (colored in blue and green, with different shades representing the FERM-SH2, pseudokinase (PK), and tyrosine kinase (TK) domains) and the nanobody stabilized complex (PDB 7T6F [S2], colored in grey).

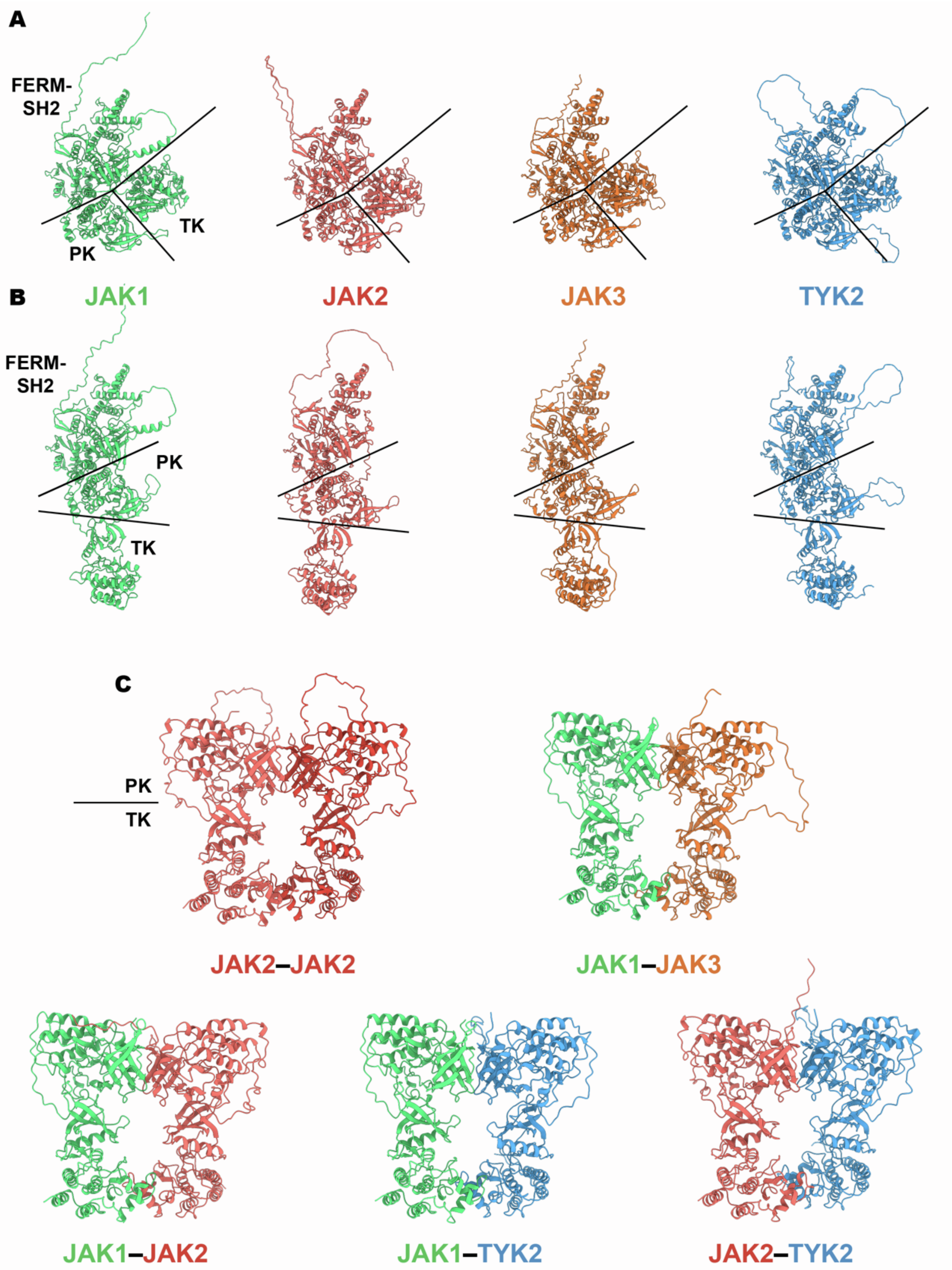

Figure S3. AlphaFold modelling of monomeric and dimeric Janus Kinases, Related to Figure 3. (**A**) Monomeric Janus Kinases in autoinhibited conformation. Human JAK1 (green, AF-A0A5F9ZI39), JAK2 (red, AF-Q506Q0), JAK3 (orange, AF-A0A024R7M7), and TYK2 (blue, AF-P29597). (**B**) Monomeric Janus Kinases in elongated conformation. Colored as in **A**. Human JAK1 (AF-P23458), JAK2 (AF-O60674), JAK3 (AF-P52333), and rat TYK2 (AF-D3ZD03). (**C**) Physiologically relevant dimeric psuedokinase (PK) and tyrosine kinase (TK) domains. Colored as in **A**.

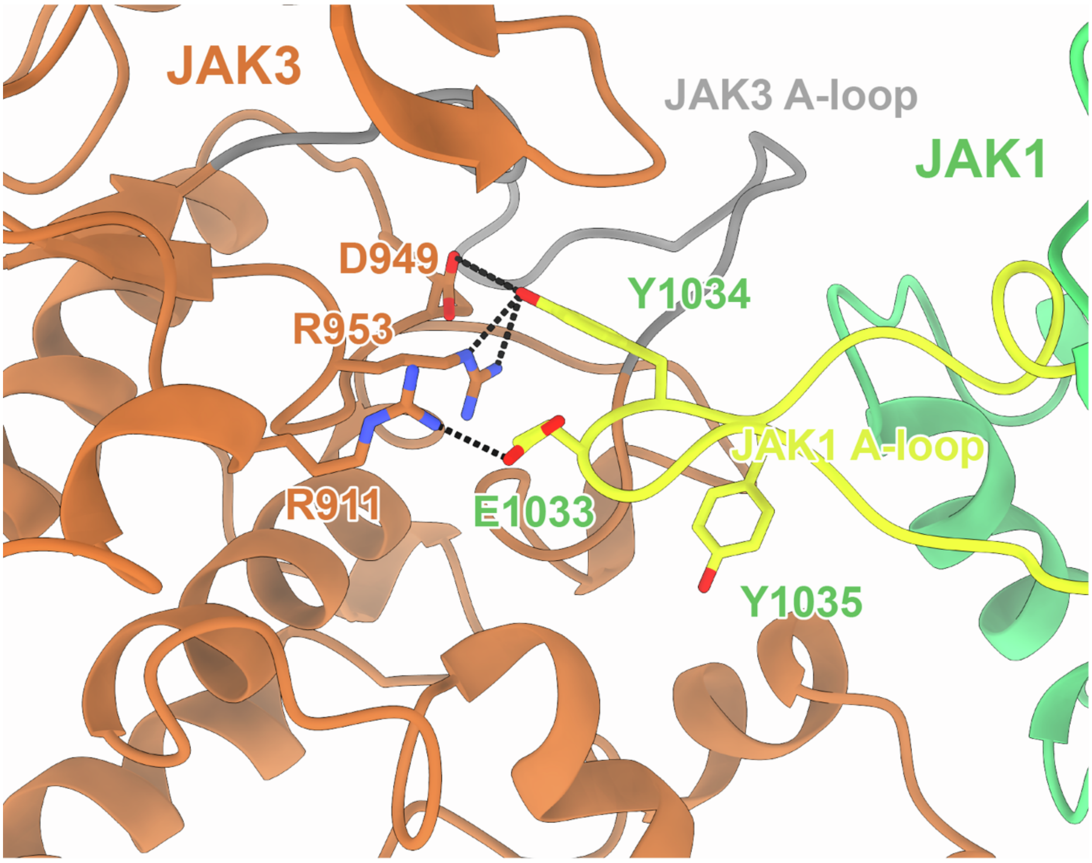

Figure S4. Modelling of A-loop rearrangement, Related to Figure 3. JAK1-JAK3 dimeric complex model with JAK1 activation (A)-loop modelled crossing to the active site of JAK3 TK. JAK1 in green, JAK3 in orange, JAK1 A-loop in yellow, and JAK3 A-loop in grey. As predicted by the modelling, Tyr1034 (P residue) in the A-loop of JAK1 is poised to hydrogen bond with Asp949 and Arg953 in the catalytic loop of JAK3, with an additional contact between Glu1033 (P-1 residue) of JAK1 and Arg911 (αD) of JAK3.

Table S1. CryoEM data collection, refinement, and validation statistics, Related to STAR Methods.

	mJAK1 Complex (PDB 8EWY/EMD-28649)
**Data collection and processing**	
Magnification	130,000
Voltage (keV)	300
Electron exposure ($e^-/Å^2$)	50.6
Defocus range (μm)	-0.8 to -3.0
Pixel size (Å)	0.653
Symmetry imposed	C2
Initial particle images	870,819
Final particle images	174,962
Map resolution FSC threshold (Å)	0.143
Map resolution (Å)	5.5
**Refinement**	
Initial model used (PDB)	7T6F/AlphaFold
Model resolution FSC threshold (Å)	0.5
Model resolution (Å)	6.3
Map sharpening *B*-factor ($Å^2$)	200
Model Composition	
Non-hydrogen atoms	10,844
Protein residues	2172
Ligands	4
B-factors ($Å^2$)	
Protein	223.41
Ligand	168.63
R.m.s. deviations	
Bond lengths (Å)	0.004
Bond angles (°)	1.076
Validation	
MolProbity score	1.26
Clashscore	1.55
Ramachandran plot	
Favoured (%)	94.78
Allowed (%)	5.13
Outliers (%)	0.09

Supplemental References

S1. Punjani, A., Rubinstein, J.L., Fleet, D.J., and Brubaker, M.A. (2017). CryoSPARC: Algorithms for rapid unsupervised cryo-EM structure determination. Nat Methods *14*, 290–296. 10.1038/nmeth.4169.

S2. Glassman, C.R., Tsutsumi, N., Saxton, R.A., Lupardus, P.J., Jude, K.M., and Christopher Garcia, K. (2022). Structure of a Janus kinase cytokine receptor complex reveals the basis for dimeric activation. Science (1979) *376*, 163–169. 10.1126/science.abn8933.
